# Sterile surgical supply waste identification using asynchronous analysis: Pediatric surgery QI pilot

**DOI:** 10.1016/j.sopen.2023.07.025

**Published:** 2023-08-09

**Authors:** Natalie M. Goldfield, Pumoli Malapati, Tyler Chafitz, Yadaven Saravanapavan, Nafisa Alamgir, Jeffrey Gander, Matthew J. Meyer

**Affiliations:** a University of Virginia School of Medicine, 1340 Jefferson Park Ave, Charlottesville, VA, USA; b Independent Researcher; c Mount Sinai Health System, 1 Gustave L. Levy Pl, New York, NY, USA; d Meharry Medical College, 1005 Dr DB Todd Jr Blvd, Nashville, TN, USA

**Keywords:** Healthcare sustainability, Operating room waste, Asynchronous analysis

## Abstract

**Background:**

The operating room (OR) is a major cost and revenue center for a hospital. One of the few modifiable costs in the OR is single-use, sterile surgical supplies (SUSSS). If SUSSS are opened on the scrub table and not used, then they are wasted. High-fidelity SUSSS usage data is important to strategically implement solutions to reduce waste of SUSSS in the OR. OR waste reduction may decrease health systems' carbon footprints and reduce spending.

**Methods:**

A convenience sample of general pediatric surgical cases was observed in summer 2021. HIPAA-free images of the surgical scrub table were acquired every 2 s with minimal impact on pediatric OR workflow. These images were asynchronously analyzed to obtain SUSSS usage data for each case.

**Results:**

Image data from three pediatric surgeons performing 41 pediatric surgeries was reviewed. The median cost of unused SUSSS was $13.10 (IQR = $2.73–$47.97) with a range of $0.07 to $489.08 wasted in a single surgery. The mean number of items wasted was 9.3 ± 6.4. The most frequently wasted items were sutures, syringes, towels, paper rulers, and specimen cups. The most expensive sources of waste were laparoscopic trocars, sutures, insufflation needles, drapes, and guidewires.

**Conclusions:**

SUSSS that were discarded without being used were successfully identified through the asynchronous analysis of HIPAA-free OR scrub table image data. This may be an opportunity to identify SUSSS waste efficiently without an observer in the OR.

## Highlights

• Single-use sterile surgical supplies are often disposed of unused after surgery.
• Sterile OR waste analysis is often limited by resource-intense data capture methods.
• Asynchronous analysis of scrub table images can identify sterile OR waste.

## Introduction

Climate change and pollution are generational threats to patients' health [1,2]. Operating rooms (OR) have a carbon footprint three to six times that of any other area in health care [3] and produce 20–33 % of a hospital's solid waste [4]. Accordingly, the OR is a major cost and revenue center for a hospital [5]. Strategically implementing solutions to reduce consumption in the OR can improve health systems' finances and reduce their impact on population health.

A major component of OR waste is single-use, sterile surgical supplies (SUSSS) such as personal protective equipment, drapes, sutures, and disposable tools. During surgery, SUSSS are opened and placed on the sterile scrub table prior to the start of a case. SUSSS cannot be reused after being opened, and are thrown out even if they were not used. Items that are opened but unused on the scrub table at the end of the case are considered “waste.”

A multicenter survey of surgeons across subspecialties estimated that 26 % of SUSSS opened for surgery remain unused at the completion of the case [6]. Hand and plastic surgeons saved over $17,000 per year by eliminating unnecessary waste from their surgical supply packs [7]. An academic neurosurgery department estimated they could save nearly $3 million if they did not open SUSSS they did not use [8].

Collecting intraoperative usage data for sterile surgical items typically relies upon either clinician memory or real time observation by knowledgeable observers. The former methodology is subjective, and the latter is resource intensive. We hypothesized waste could be identified through asynchronous review of intraoperative scrub table images.

We designed a quality initiative to collect HIPAA-free scrub table images from pediatric general surgical operations and analyze the images offsite to identify opened SUSSS and their usage. Our aims were: 1) to test if scrub table images could be used to identify unnecessary waste, 2) to understand the magnitude of cost of waste in pediatric surgery, 3) to identify which items were most commonly wasted across various procedures, and 4) to identify any trends that could inform anticipated quality initiatives to reduce the unnecessary waste of SUSSS at our institution.

## Methods

We designed a process to collect SUSSS usage data that could be reviewed with minimal impact upon OR processes. The division of Pediatric Surgery was selected for our initiative because they perform a wide variety of cases (some with limited amounts of SUSSS for simplicity in the pilot), and were interested in analyzing their SUSSS usage. Specific attention was directed towards avoiding HIPAA-protected data. Elective pediatric surgery cases were collected as a convenience sample during June and July 2021.

### Intraoperative workflow and data extraction

**Fig. 1:**
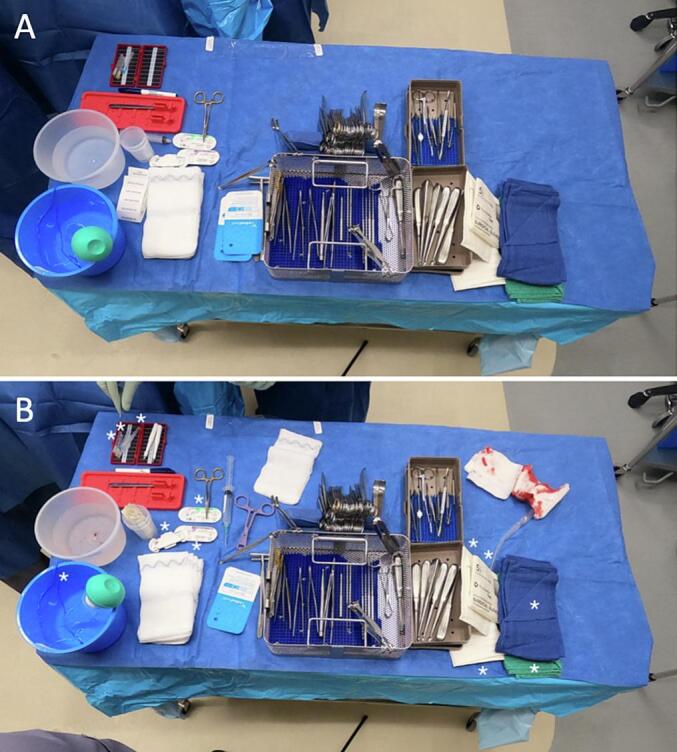
Images of SUSSS and instruments on a surgical scrub table. Image (A) depicts a sterile scrub table at the start of a pediatric surgery case for a cyst. Image (B) depicts the same table at the end of the case with numerous unused (wasted) sterile supplies including gloves, sutures, towels, and sponges.

Prior to the start of each case, a *GoPro Hero 8* (San Mateo, CA, USA) camera was carefully mounted on a tripod behind the scrub table. The camera was configured so neither patient nor patient-identifying information was recorded (Fig. 1). Once the operation began, the camera would take an image of the scrub table every 2 s until the procedure was completed–image capture was confirmed using the *GoProQuik* application. After the operation, the image files of the scrub table were securely stored on *Box* (Redwood City, CA, USA).

Images from each surgery were analyzed frame by frame (NG) for SUSSS usage and spot-checked for quality control (TC). SUSSS identified in the first image of the case was used as a baseline to quantify SUSSS opened during the surgery. Each subsequent photo was reviewed for the addition, usage, or removal of SUSSS. If SUSSS was added to the scrub table, it was included in the baseline quantification of SUSSS. SUSSS that left the field of view were considered used. Once all images were reviewed, the SUSSS remaining on the scrub table were considered unused. Data was compiled into *Google Sheets* (Mountain View, CA, USA).

### Measures

Measures were focused on identifying financial and physical waste; defined as SUSSS that are opened on the scrub table but unused during surgery. The primary measure was financial waste defined as the cost of opened but unused SUSSS remaining on the scrub table at the end of surgery. Secondary measures were the number of individual SUSSS wasted per surgery, percent of financial waste of total cost, and percent of items wasted.

### Statistical analysis and ethical considerations

Descriptive data was presented using median and interquartile range for financial data, and mean and standard deviation for accounting of SUSSS physical waste. Nonparametric analyses were used for financial data comparisons, and parametric analyses were used for comparing the accounting of SUSSS physical waste. ANOVA was used to analyze association between surgeon and cost and amount of SUSSS waste. Linear regression was used to analyze the relationship between length of surgical case and cost and amount of SUSSS waste. Analyses were performed using IBM SPSS (28.0.1.1). The protocol for capturing scrub table data was reviewed by the UVA Health IRB for Health Sciences Research and considered to be a quality improvement initiative. Data was collected in three pediatric surgery operating rooms at UVA Health, a tertiary, academic referral center in Charlottesville, Virginia, USA.

### Financial data capture and estimates

Cost data for items was provided by UVA Health Supply Chain Management. Cost data denotes amount paid to the supplier by the hospital system for each item. Costs of specific items in a surgical kit were obtained. Exact prices of individual items are not reported.

There were seven items for which we were unable to capture specific institutional costs: four we estimated using similar items, and two we estimated using online marketplaces. Surgical suture was the last item for which we did not use specific institutional costs. Due to the breadth and number of sutures used during the cases, we did not document the specific type, just the number of sutures. For all sutures, except the rare speciality suture with an easily identifiable specific price, we used the median cost of sutures in our inventory rounded to the nearest whole dollar ($5).

## Results

### Cases observed

Image data from three pediatric surgeons performing a total of 44 operations were collected from June to July 2021. Three cases were excluded due to inadequate imaging of the scrub table. The mean operative time was 69 min (range: 12 to 193 min). Cases varied from common pediatric surgeries like gastrostomies, hernia repairs, and port-a-cath placements, to more complex such as pulmonary lobectomies and intestinal fistula repairs (Table 1).

**Table 1:** Median surgical supply cost and mean number of surgical items that were opened but unused stratified by pediatric surgery procedure. *Range rather than IQR. **Mean as there are only two data points. IQR: interquartile range. STDEV: standard deviation.

	Cases	Cost of Wasted SUSSS Median (IQR)	Waste as Percent of Cost of Opened SUSSS Median (IQR)	Number of SUSSS Wasted Mean (STDEV)	Waste as Percent of Opened SUSSS Mean (STDEV)
ALL	41	$13.1 (2.73-$47.97)	8.6 % (2.9–27.3 %)	9.3 (6.4)	26.2 % (14.8)
Gastrostomy	11	$4.07 (1.64-$12.02)	3.4 % (1.0–21.6 %)	6.4 (5.9)	21.4 % (16.7)
Cholecystectomy	4	$10.05 (1.12-$20.53)	1.5 % (1.0–3.1 %)	6.8 (2.8)	18.9 % (3.2)
Hernia Repair	4	$3.44 (1.09-$165.58)	7.9 % (2.5–40.3 %)	6.5 (4.7)	22.6 % (9.7)
Nuss	4	$42.47 (19.81-$61.32)	17.1 % (12.2–23.7 %)	11.0 (5.3)	23.0 % (6.1)
Cyst	3	$18.65 (1.38-$55.78)^⁎^	47.2 % (8.6 %–92.5 %)^⁎^	10 (7.55)	43.9 % (26.73)
Port	3	$1.82 (1.22-$35.50)^⁎^	4.34 % (4.2 %–34.2 %)^⁎^	6 (4.58)	18.37 % (7.76)
Orchiopexy	2	$271.64^⁎⁎^ (54.21-$489.10)^⁎^	57.0 %^⁎⁎^ (21.0 %–93.0 %)^⁎^	16.50 (10.61)	44.5 % (21.92)
Appendectomy	1	$13.10	3.0 %	9	28.1 %
Enterostomy	1	$10.49	24.1 %	29	46.8 %
Enteroenterostomy	1	$16.69	13.2 %	14	28.0 %
Esophagomyotomy	1	$18.47	2.9 %	10	21.7 %
Foreign Body Removal	1	$3.65	42.3 %	6	33.3 %
Gastrectomy	1	$193.95	33.3 %	10	25.6 %
Gastrostomy + Port	1	$45.27	17.2 %	18	45.0 %
Ileostomy	1	$58.07	14.9 %	14	24.1 %
Intestinal Fistula	1	$56.74	39.0 %	15	39.5 %
Lobectomy	1	$16.40	0.5 %	7	13.7 %

### Total unused SUSSS and costs

**Fig. 2:**
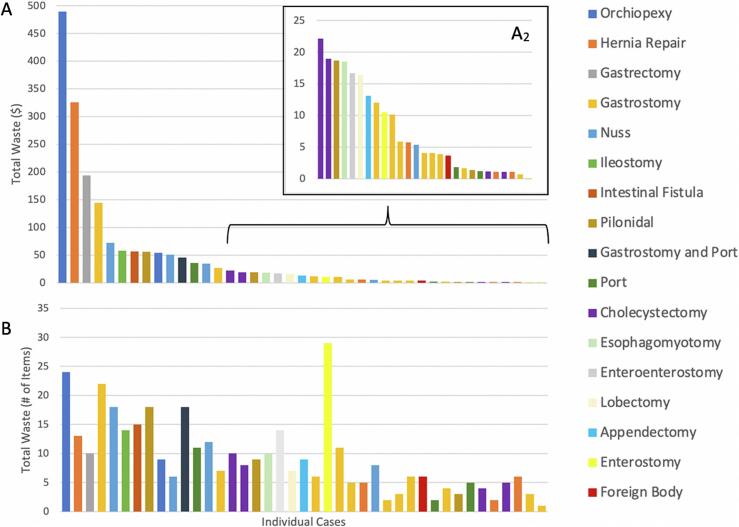
Total cost of SUSSS wasted and total number of SUSSS wasted across 41 pediatric surgery cases at UVA in June–July 2021. Cases are arranged from highest to lowest cost of waste in both panel (A) and (B). The color of each bar graph corresponds to the type of surgical case. The nested graph (A2) depicts cases with less than $25.00 of waste in descending order for better visualization.

The median cost of unused SUSSS was $13.10 (IQR = $2.73–$47.97) and the mean number of items wasted was 9.3 ± 6.4 (Fig. 2). The total cost of wasted supplies across all surgeries was $1842.53, with a range of $0.07 to $489.08 wasted in a single surgery. The cost per unit of waste was $4.85 (total identified cost of waste from all 41 surgeries divided by total units of items wasted from all surgeries).

The median baseline cost of SUSSS open and available on the scrub table was $141.54 (IQR = $47.21–$501.78). The mean number of those items was 34.4 ± 12.7. The mean percentage of wasted surgical supplies across all surgeries was 26.2 % ± 14.8 % with a range from 4 to 72 %.

### Most frequently wasted supplies

The most frequently wasted items were sutures, syringes, towels, paper rulers, and specimen cups. These items accounted for 247 of the 380 wasted SUSSS documented (Table 2). The most expensive sources of waste were laparoscopic trocars, sutures, insufflation needles, drapes, and guidewires. This grouping of expensive items alone accounts for $1551.94 of the $1842.53 of total wasted cost.

**Table 2:** Most frequently wasted and most expensive wasted SUSSS in pediatric surgery. *Includes three different trocars. **Includes four different drapes.

Most frequently wasted supplies	Total number opened	Total number wasted
Sutures	358	87
Syringes	290	87
OR blue towel	66	33
Ruler	65	25
60cc Cup	73	15


Most expensive wasted supplies	Total cost opened	Total cost wasted
Trocars^⁎^	$5706.39	$728.05
Sutures	$1893.82	$460.23
Insufflation Needle	$604.03	$181.26
Drapes^⁎⁎^	$93.02	$91.90

### Predictors of SUSSS waste

The surgeon operating was neither a significant predictor of the cost of wasted supplies in a given procedure (*p* = 0.733) nor number of items wasted in a given procedure (*p* = 0.901). Length of surgery predicted neither cost of wasted items (*p* = 0.81) nor total number of SUSSS wasted (*p* = 0.054).

## Discussion

Our asynchronous analysis of pediatric surgery scrub table images demonstrated it is possible to identify SUSSS waste without making real-time observations in the OR. The total cost of unused supplies from all 41 surgeries included in the analysis was $1842.53 with the cost of waste in a single surgery ranging from $0.07 to $489.08. Suture and syringes were the items most commonly wasted. We know of no other study that identified waste of SUSSS through asynchronously analyzed HIPAA-free scrub table images.

### SUSSS waste: cost of items

The median cost of SUSSS waste in the pediatric OR was $13.10. This is similar to the average cost of SUSSS waste in Welsh otolaryngology cases ($8.76) [9], and French urological, gynecological and digestive cases (€1.4–6.1) [10]. The range of SUSSS waste cost ($0.07 to $489.08) is similar to that of general surgery procedures at a military medical center ($0 to $486.43) that were identified through direct observation [11]. These similarities support that our methodology of asynchronously evaluating scrub table imaging alone may be sufficient to identify SUSSS waste.

Notably, the average cost of SUSSS waste in our study is lower than that from the UCSF Health Department of Neurosurgery which found an average of $653 per case of opened but unused supplies [8]. This is likely due to the difference between the supply costs of subspecialties. Neurosurgical, along with cardiac and orthopedic, cases have been identified as surgical subspecialties with high costs of wasted supplies [12].

The items most frequently wasted in our study were sutures, syringes, and towels. These items are ubiquitous across procedures and specialties and may be generalizable waste reduction targets. Suture, specifically, is an opportunity for cost-savings. In our model, we used the mean cost of suture in our institution ($5). However, suture needles have a wide range of prices ($1 to >$50 per suture) and it is very possible that the actual cost of unnecessary suture waste was larger than we calculated. Educating surgeons on the cost of items, such as suture needles, has been shown to reduce waste [13]. More expensive items, such as trocars, insufflation needles, and guidewire kits, were seldom opened and unused, but when they were unused made-up a large fraction of the total cost of SUSSS waste.

Our results document $1842.53 of SUSSS waste in 41 cases. The pediatric surgery division performs approximately 1100 surgeries per year. If the operations in our sample are representative, we calculate the pediatric surgery division generates almost $50,000 in waste of SUSSS each year. Using supply chain greenhouse gas factors for “surgical and medical instruments” and “surgical appliance and supplies manufacturing,” we calculate 10 tons of carbon dioxide emissions from wasted supplies in our pediatric ORs alone [14].

Considering the brevity of many of the pediatric surgery cases we assessed, we expect the cost of waste to be equal or greater in most other surgical subspecialties [12]. On average and across specialties, surgical supplies cost $3 per surgical minute [15]. In our tertiary, academic medical center with over 30 operating rooms, we estimate SUSSS waste to cost approximately $3 million dollars each year. When SUSSS waste is extrapolated to the estimated 64 million surgeries performed annually in the US [16], it is in the billions of dollars.

### SUSSS waste: amount of items

In the pediatric ORs, 26 % (*n* = 1415) of SUSSS open on the scrub table at the start of surgery were discarded without being used. This is almost exactly the amount surgeons at our institution estimated (27.3 %) when asked “the percentage of SUSSS that were opened but unused at the end of surgery.” [6] However, compared to general surgical cases at a military hospital (average 8.3 % SUSSS wasted), our 26 % average was the high end of their range (27 %) [11].

This difference is likely attributable to a key methodological difference. Given our effort to asynchronously analyze waste through images, our baseline count of SUSSS (denominator in the waste equation) is the sum of only those items remaining on the scrub table after prepping and draping the patient. Compared to studies including all items for the surgery including prepping, draping, and SUSSS starting on the mayo stand, our methodology likely has a smaller denominator and consequently results in a higher percentage of SUSSS wasted.

In our study, the specific surgeon operating predicted neither cost of waste nor number of items wasted. A recent review of pediatric surgeons across divisions similarly found neither correlation between individual surgeon and cost nor case volume, years in practice or length of surgery [17]. A five-year assessment of OR waste from four colorectal surgeons identified only 0.89 % of sterile supplies and 0.59 % of sutures were documented as opened but unused [18]. This supports that a well-designed system can reduce SUSSS waste to a nearly negligible amount.

### SUSSS waste: impact and root causes

Removing waste from the OR and healthcare can contribute to improving public health outcomes. Physical healthcare waste is often disposed of in energy-intensive ways such as incineration. Incineration releases harmful pollutants into the environment and is associated with oncologic and pulmonary pathology as well as congenital abnormalities [19], and in the US tends to harm environmental justice communities the most [20]. The need for high-quality research to guide sustainable practices in healthcare is critical, as patient health is inclusive of both direct patient care that occurs in the OR and indirect impact of OR waste on the environment in which patients live.

Previous work identifies several barriers for overcoming OR waste: lack of awareness of the issue, lack of environmental and carbon footprint considerations, lack of time to address the issue, physician preference, and perceived safety benefit [6,21]. The latter barrier of patient safety is a common and compelling argument towards opening items preemptively: procedure delays to retrieve surgical items pose an inherent risk to patient safety in the case of urgent events. However, the most often discarded items in our study (e.g. syringes, towels, paper rulers) are notably inexpensive, common, and seldom used emergently. Sutures, though sometimes used urgently, would often need to be pulled by a circulator in the case of unexpected surgical events. Items that are more expensive and less frequently wasted (e.g. trocars, insufflation needles, drapes) are similarly not used reflexively during adverse events.

Other concerns for patient safety include prolonged OR or anesthesia time, a complex trade-off that merits investigation. Though prior studies have demonstrated reduced tray preparation time with removal of unnecessary instruments [22], change in OR time has yet to be investigated. Despite these significant barriers, we are encouraged by the strong consensus on this issue: 90 % of surgeons and OR personnel agree that OR waste is excessive [6,21] and 95 % of surgeons are willing to change OR workflow to reduce waste [6].

### Reducing SUSSS waste

Initiatives to decrease waste in the operating room have been successfully implemented on a small scale and align on common themes of feedback and education. Several initiatives that decreased spending after implementation included periodic cost scorecards to surgeons [13,23], real-time surgeon specific feedback on operative expenditures [[24], [25], [26]], and an educational program for surgeons on their intraoperative waste [27]. These studies provide encouraging evidence that surgeons can reduce perioperative waste if provided with actionable information.

One waste reduction study focused on pediatric laparoscopic surgeries. By comparing the surgeons' costs to each other and offering suggestions for lowering costs, this initiative was able to reduce the median cost per case by $190 without impacting their clinical outcomes [23]. This cost-savings of $190 is much higher than the SUSSS waste identified in most of our cases, and demonstrates there is an opportunity to eliminate even more SUSSS cost and waste by engaging with surgeons' SUSSS usage in a clinically nuanced manner.

At our own institution, we employ standardized preference cards for each procedure listed. These preference cards are periodically reviewed and modified to align with surgeons' preferred supplies. Supplies are selected and opened by the scrub technician and circulating nurse prior to the surgeon entering the operating room. Items on preference cards may have comments attached instructing they not be opened unless specifically requested by the surgeon.

These efforts to reduce perioperative waste still result in analysis identifying 26 % of items open on the scrub table at the start of surgery never being used. In a recently published survey of surgeons, commonly cited reasons for witnessed OR waste included miscommunication (75 % of respondents), sterile surgical kits (66 %), and overpreparation for possible emergencies (60 %) [6]. To further reduce waste at our institution, we recommend the following strategies: 1) increasing the frequency and ease of preference list revision; 2) holding more items off the sterile field until needed, especially sutures and high-value items; 3) price transparency for surgical supplies; and 4) education about OR waste to surgical teams. Through enhanced preoperative planning and engagement of staff in waste reduction initiatives, we see the potential to address the aforementioned root causes.

Dr. Atul Gawande's *New Yorker* article “What Big Medicine Can Learn from the Cheesecake Factory” stated “…[Cheesecake restaurant] managers aimed at throwing away no more than 2.5% of the groceries they bought…” The OR wasting 26 % of SUSSS, or 10 times the acceptable amount at the Cheesecake Factory, seems indefensible in such a highly controlled environment. Unquestionably some intraoperative SUSSS waste is necessary: preparation for the high probability events that may not happen, or low probability catastrophes that need immediate intervention. Frequent monitoring of the scrub table with consistent identification of SUSSS waste may provide essential data to perioperative administrators, allowing them to objectively eliminate unnecessary SUSSS waste without affecting patient outcomes. Even a small amount of waste has a large impact when scaled to the magnitude of the global healthcare system.

## Limitations

Our study, like many high-fidelity OR waste observations, had a small sample size. Emergent, weekend, and night-time cases were not included in this analysis. Waste may have been underestimated, as there are times when SUSSS was “used” simply because it was available and not because it was necessary (e.g. using additional suture because it was on the scrub table). We neither recorded nor analyzed the mayo stand or any other back table used for preparation. Recording began when the scrub table was moved into position for the start of surgery and ended when drapes were taken down. Therefore, items used prior to positioning of the scrub table, including drapes, gowns, and gloves, are not reflected in this dataset. Finally, all surgeons and OR personnel were aware of the quality improvement initiative, and a Hawthorne Effect biasing both towards conservation and utilization of SUSSS is possible.

## Conclusion

SUSSS waste in the OR places a financial and environmental burden on our healthcare system. Our pilot used asynchronously analyzed image data of the HIPAA-free OR scrub table to document a nontrivial amount of SUSSS that were discarded without being used, making this an opportunity for cost-saving and waste reduction. One of the suggested barriers to reducing this source of waste is a paucity of data on opened but unused surgical supplies. Specific and individualized data on sources of waste may help health care systems implement targeted interventions to decrease OR waste. Given the rising costs of healthcare and immeasurable global impacts of waste and incineration on our environment, it is imperative that we continue to investigate perioperative waste such that novel strategies for waste reduction may be employed in the healthcare sector.

## CRediT authorship contribution statement

MM conceptualized and designed the study, coordinated data collection, assisted in data analysis, and wrote first and subsequent drafts of the manuscript. NG collected and organized data, conducted data analysis and wrote first and subsequent drafts of the manuscript. PM conceptualized and designed the study and wrote first and subsequent drafts of the manuscript. TC conceptualized and designed the study, participated in data organization and analysis, and critically reviewed and revised the manuscript. YS participated in data collection and analysis and critically reviewed and revised the manuscript. JG conceptualized and designed the study, coordinated data collection, and critically reviewed and revised the manuscript. All authors approved the final manuscript and agreed to be accountable for all aspects of the work.

## Funding sources

We received support for our research from the integrated Translational Health Research Institute of Virginia (iThriv) which is funded through the National Center for Advancing Translational Science of the National Institutes of Health Award UL1TR003015/KL2TR003016.

## Ethics approval

Upon review by the University of Virginia Health Science Research IRB, this work was deemed quality improvement.

## Declaration of competing interest

We declare that this manuscript is original, has not been published before, and is not being considered for publication elsewhere. This study was presented in part at the 2022 Planetary Health Annual Meeting. Matthew J. Meyer, Pumoli Malapati, Tyler Chafitz, and Nafisa Alamgir have interest in University-owned intellectual property related to technology that assists in the identification of intraoperative sterile surgical waste. They have stock in a company that may one day license this technology. This study was supported by a grant from the integrated Translational Health Research Institute of Virginia (iThriv) which is funded through the National Center for Advancing Translational Science of the National Institutes of Health. Generative-AI has NOT been used in the creation of this manuscript.

As corresponding author, I confirm that the manuscript has been read and approved for submission by all named authors.
